# Methodological developments in qualitative longitudinal research: the advantages and challenges of regular telephone contact with participants in a qualitative longitudinal interview study

**DOI:** 10.1186/s13104-015-1107-y

**Published:** 2015-04-11

**Authors:** Emma Carduff, Scott A Murray, Marilyn Kendall

**Affiliations:** Primary Palliative Care Research Group, Centre for Population Health Sciences, The Usher Institute of Population Health Sciences and Informatics, The University of Edinburgh, Medical School, Teviot Place, Edinburgh, EH8 9AG, UK; Marie Curie, Fairmile, Frogston Road West, Edinburgh, EH10 7DR, UK

**Keywords:** Cancer, Death and dying, Interviews, End of life issues, Palliative care, Qualitative longitudinal research, Research design, Research relationship, Telephone interviews

## Abstract

**Background:**

Qualitative longitudinal research is an evolving methodology, particularly within health care research. It facilitates a nuanced understanding of how phenomena change over time and is ripe for innovative approaches. However, methodological reflections which are tailored to health care research are scarce. This article provides a synthesised and practical account of the advantages and challenges of maintaining regular telephone contact between interviews with participants in a qualitative longitudinal study.

**Methods:**

Participants with metastatic colorectal cancer were interviewed at 3 time points over the course of a year. Half the group also received monthly telephone calls to explore the added value and the feasibility of capturing change as close to when it was occurring as possible.

**Results:**

The data gathered from the telephone calls added context to the participants’ overall narrative and informed subsequent interviews. The telephone calls meant we were able to capture change close to when it happened and there was a more evolved, and involved, relationship between the researcher and the participants who were called on a monthly basis. However, ethical challenges were amplified, boundaries of the participant/researcher relationship questioned, and there was the added analytical burden.

**Conclusions:**

The telephone calls facilitated a more nuanced understanding of the illness experience to emerge, when compared with the interview only group. The findings suggest that intensive telephone contact may be justified if retention is an issue, when the phenomena being studied is unpredictable and when participants feel disempowered or lack control. These are potential issues for research involving participants with long-term illness.

## Background

Qualitative longitudinal research has an established role in social science disciplines such as anthropology, criminology, education, psychology, social policy, and sociology. Contemporary qualitative longitudinal research is driven by a desire to understand what change happens, but also how and why it happens, in the socio-cultural context [1]. It also captures the interplay between time, and the cultural dimensions of social life, thus depicting time as non-linear [2]. One of the major advantages of qualitative longitudinal research is the nuanced understanding of phenomena which evolves through time. This is particularly relevant for understanding the experience of illness.

One key facet of qualitative longitudinal data generation is that it is iterative, drawing on what was learnt previously to understand what has changed to tell a story over time [3]. Qualitative longitudinal studies are complex, and there are many variables in terms of study design, including the length. Saldana argued that length is dependent on context, theoretical paradigm and discipline [4]. So too is the question of when to generate data throughout the longitudinal study, although our review of the literature showed that this is rarely discussed.

Longitudinal research is about exploring change, but what changes occur (if any) are dependant on the context of the study. Lewis highlights the multi-faceted nature of change that can be exposed through analysing qualitative longitudinal data in evaluation research: 1) Narrative change shows how individual stories change over time, 2) Change in the context of time, when a story is retold and re-interpreted by the participant at later interviews, 3) Change that is evoked as the researcher sees the participant more clearly over time, 4) In some cases there will be no change [5]. The qualitative longitudinal researcher should confront their preconceptions about change and let a definition of change (if there is one) emerge [4-6].

The potential benefits of a qualitative longitudinal approach for exploring the needs of people with progressive diseases have been highlighted in response to the complex, temporal changes that these people experience [7-9]. Qualitative longitudinal research in health care has increased in popularity over recent years [10-15], yet there are still relatively few studies when compared to one-off qualitative studies. Possible reasons for this include: financial constraints from funders who privilege quantitative methods more than qualitative, concerns about recruiting people suffering long-term illness to longitudinal studies [8], and the epistemological difference between the social sciences and medicine [16], insomuch as health care research remains a predominantly positivist discipline, where randomised control trials and cohort studies are considered to be the gold standard. When compared with social science journals, the short word limits in many health care journals might prevent qualitative longitudinal researchers adequately detailing and reflecting on their methods and the complex issues that they might face. Nevertheless, there is growing recognition of the need for methodological literature which is tailored to the complexities of conducting qualitative longitudinal health care research [1,10].

This paper aims to expand the methodological discussion of qualitative longitudinal research. We provide a synthesised and practical account of the benefits and challenges of telephoning participants between face to face interviews in a qualitative longitudinal study. This paper extends from a study which was designed to explore the overall potential of qualitative longitudinal methods for understanding the experience of metastatic colorectal cancer.

## Methods

We adopted a qualitative longitudinal design using interviews and telephone calls with participants with metastatic colorectal cancer to generate data over the course of 1 year. The design involved 2 groups of participants; in group 1 (6 monthly group), interviews were conducted at regular intervals of 6 months and no other contact was made between the waves of data generation (number of participants = 8). We decided on 6 monthly intervals for 1 year considering the average prognosis of our participants [17]. This decision was guided by advice from specialist colorectal cancer physicians and previous end-of-life qualitative longitudinal studies which involved patients with similar prognoses. In group 2 (flexible interval group), interviews were supplemented by monthly telephone calls (number of participants = 8).

### Recruitment

Participants with metastatic colorectal cancer (Dukes stage D) were recruited from the outpatient clinic of a national cancer centre in Scotland and were randomly assigned to each group. Ethical approval was granted by the South East Scotland Research Ethics Committee. The study was introduced to participants at the time of their consultation with the physician. If they were willing, the researcher (EC) explained the study, gave them a participant information sheet and agreed to telephone them a few days later to discuss the study. Written informed consent for participation in the study was obtained from participants prior to the first face to face interview. Verbal informed consent was obtained prior to each subsequent interview.

### Data collection

This study adopted a narrative approach to interviewing and analysis. Narratives are commonplace in medicine and there is increasing awareness that those with illness tell stories; to their doctor, their family, their friends, and in this way they recapitulate events [18-20]. It has been argued that people make sense of their illness through narratives [21] and that studying them can transform stories of individual experience of illness to a social phenomenon [22]. We hypothesised that those with advanced colorectal cancer would have a story to tell and that narrative methods would encourage participants to share their accounts. The researcher (EC) commenced each interview by asking the participant to describe what had happened to them, meaning they could start their story where they wanted. All interviews were audio-recorded and transcribed by a paid transcriber, and then checked by the researcher. Each participant was assigned a pseudonym.

Telephone conversations are commonly used as a mode of social interaction and are not socially or age exclusive [23]. We conducted telephone calls with each of the 8 participants in the flexible group on a monthly basis. Although participants had the researcher’s contact details on the information sheet they had received, we did not encourage them to call, and received no telephone calls over the course of the study. The telephone calls to the participants were not digitally audio-recorded, because we hoped that the informality of the telephone conversations, in comparison to the face to face interviews, would allow the relationship between the researcher and the participants to flourish [23]. Instead, detailed field notes were written immediately after the telephone calls [24]. The participants were aware that the telephone calls would be used as data for the study.

During the calls, participants were asked to consider how their situation had changed. If there had been a significant change, they were invited to meet for an interview before the default 6 month interval. If no change was identified, we met again no more than 6 months after the previous interview to a maximum of 3 interviews. We used Thomson et al’s definition of critical moment - an event described in an interview which either the researcher or interviewee thinks will effect lives or identities [25]. We adopted this definition because we thought it was broad enough to capture change in all 4 domains of the illness experience; physical, psychological, social and spiritual [25].

### Analysis

We used a narrative analysis using the Voice Centred Relational Method (VCRM) [26]. Other methods of narrative analysis are more prescriptive, and some have been criticised for fragmenting and decontextualizing narratives [20,27,28]. VCRM is an inclusive method, which focuses on what is said by participants but also what is unsaid through 4 different readings of each interview transcript.

- Reading 1a is for overall plot; what story is the participant telling? Who are the characters? What events which unfold over the course of the story?
- Reading 1b invites the researcher to share thoughts and feelings about the interview.
- Reading 2 focuses on the active voice of the participants, and how they used pronouns throughout the narrative.
- Reading 3 explores the participants’ relationships and how they spoke and saw themselves in relation to others.
- Reading 4 is about context, and the wider social and cultural frameworks in which the stories are told and heard [26].

One of the advantages of qualitative longitudinal research is the multiple opportunities that it presents in terms of analysis. The data were analysed; for each individual at each time point, as patient groups at each time point, and longitudinally, where individual cases were examined over time.

## Results

Tables 1 and 2 show the demographics of groups 1 and 2, respectively. The sample comprised of 10 men and 6 women, with a median age of 64 years (range 48 to 80 years). The sample size allowed the researcher to maintain engagement with the vast amount of data. The median time from diagnosis of the metastases to recruitment into the study was 9 months (range 2 to 18 months). All the participants had been told that the cancer could not be cured prior to recruitment. Some were receiving palliative treatment, others were not. Eight of the patients identified a family member who was considered to have a caring role and they too were recruited to the study.

**Table 1:** **Characteristics and number of interviews for participants in group 1 (6 monthly group)**

**Participant pseudonym**	**Age at recruitment (years)**	**Diagnosis (primary cancer/metastases)**	**Months from diagnosis of metastatic disease to recruitment**	**Interviews conducted Time point, BER = bereavement**	**Status at study end**
Ann	67	Colon/peritoneal metastases	2	T1, T2, T3	Surviving
Brenda	60	Colon/liver metastases	6	T1, T2BER, T3BER	Deceased
Andrew	57	Colon/liver metastases	9	T1, T2, T3	Surviving
Brian	59	Colon/liver and lung metastases	<18 months	T1, T2, T3	Surviving
Cath	48	Colon/liver and peritoneal metastases	15	T1, T2	Hospice, T3 abandoned
Chris	80	Colon/lung and liver metastases	13	T1, T2, T3	Surviving
Duncan	59	Rectal/liver metastases	10	T1, T2	Deceased
Edward	74	Colon/peritoneal metastases	7	T1	Deceased

**Table 2:** **Characteristics, number of interviews and number of telephone calls for participants in group 2 (flexible interval group)**

**Participant pseudonym**	**Age at recruitment (years)**	**Diagnosis (primary/metastatic)**	**Months from diagnosis of metastatic disease to recruitment**	**Interviews conducted time point, BER = bereavement**	**Number of telephone calls**	**Status at study end**
Fred	73	Colon/lung and liver metastases	13	T1	5	Deceased
Gordon	65	Colon/liver metastases	6	T1, T2, T3	6	Surviving
Deirdre	66	Colon/cervical and vaginal metastases	5	T1, T2	7	Deceased
Harry	76	Colon/liver metastases	12	T1, T2, T3BER	7	Deceased
Eve	62	Colon/lung and liver metastases	6	T1, T2	2	Deceased
Ian	65	Colon/liver metastases	2	T1, T2, T3	9	Surviving
John	62	Colon/liver metastases	11	T1, T2, T3BER	5	Deceased
Faye	55	Colon/lung and liver metastases	9	T1, T2, T3	5	Surviving

Table 3 shows the number of interviews conducted at each time point and the total number of telephone interviews. Thirty-six interviews were conducted in total, each lasting between 30 minutes and 120 minutes. Generally, the interviews became shorter as the study progressed. Two participants requested that the interviews be conducted at the hospital. The telephone calls totalled 46 by the end of the study and ranged in length from just a few minutes to approximately 40 minutes. All attrition resulted from death.

**Table 3:** **Number of interviews conducted at each time point for patients and total number of telephone interviews**

	**Time 1**		**Time 2**		**Time 3**	
	**6 months**	**Flexible**	**6 months**	**Flexible**	**6 months**	**Flexible**
**Patient interviews**	**8**	**8**	**6**	**7**	**4**	**3**
**Total number of patient interviews**	**16**		**13**		**7**	
**Total number of interviews**	**36**					
**Total number of telephone calls**	**46**					

The results are supported by excerpts from the researcher’s (EC) field notes, the monthly telephone calls and in some instances quotes from the face to face interviews.

### Advantages of monthly telephone calls

#### Contextualising the narrative

The data gathered from the telephone calls added contextual information to the accounts of illness. We gained a real-time impression of how the illness progressed, and were able to map those ‘ups and downs’, which the participants in this study described as characteristic of the experience of advanced cancer. The longitudinal analysis explored how the contextual aspects of the illness stories changed over time. For example, Deirdre ceased discussing her friends or talking about social outings during our telephone calls. She did not seem to be socialising much at all; an activity she had previously enjoyed. At the subsequent face to face interview, Deirdre told the researcher that she had not seen her friends for some time. Another participant, Fred, explained that he and his wife had decided to take a day trip for some respite. This was something they had enjoyed before the illness and describing it enabled us to understand how he was striving to maintain some aspects of his pre-illness lifestyle. John mentioned he had given up his much-loved hobby during the third telephone call; a longstanding pastime that he had enjoyed for decades. We asked John to reflect on his feelings about this at interview 2 and whether it had affected his quality of life. In this way, the contextual data from the telephone calls also informed subsequent interviews.

#### Capturing change as it happened

The telephone conversations gave participants the opportunity to consider critical events or junctures in the illness trajectory that were significant to them. For example, during the fourth telephone call, Deirdre mentioned that she would discontinue chemotherapy if she suffered the same hideous side effects that had resulted in an earlier hospitalisation. At the time, we were unsure of the significance of her telling this within her overall narrative, but were given the opportunity to explore the issue further at a face to face interview a month later. During the longitudinal analysis of Deirdre’s case study, we identified that her thoughts about discontinuing treatment signified her strive to ensure that the responsibility for making decisions about her care remained her own. Although we originally thought that this signified her desire to maintain autonomy, the longitudinal analysis showed that it was actually about her family. Deirdre did not want them to be distressed by the decisions they felt they had to make on her behalf, as the quote below illustrates.

“I says ‘I don’t want [husband] or [daughter] and [son] to have to make the decision to pull my plug’. I’m saying now that if it’s a no-go thing, then just do it.” (Deirdre, interview 2)

Using data from the telephone calls informed the topic guide for subsequent interviews and enabled us to track the process of change that participants described.

John described how he had asked his doctor about his prognosis at telephone call 2. We considered this to be a critical moment as he not asked before, and as a result, John started to plan the remaining time he had. Below is an excerpt from the researcher’s field notes which were written immediately after the second telephone call with him. John had not asked about his prognosis at interview 1. Indeed Mary, John’s sister, who was also recruited to the study, confided to the researcher that John had thought he must have at least a year to live because he had been invited to participate in this longitudinal study. This suggests he had previously considered his prognosis which supported the notion that John asking about it at month 2 was significant.

John has had what he described as a strange week. He watched a film about 2 men, both with cancer, who met in hospital and decided to write a list of things they wanted to do before they died. John found the movie really poignant, and for him it was the impetus to ask about his prognosis when he went to clinic yesterday. Both Mary (John’s sister) and John had told me that they hadn’t yet inquired about this let alone talk about the future and what may happen. I think he had been building up to this for some time and the film was the catalyst. So he asked the doctor yesterday about how long he had left and was told it would probably be months, rather than years, but could even be 12 – 24 months. I asked him how he felt about this and he responded that he really didn’t have any idea how long it would be, so in a way it was a relief to know. Knowing this information also made him think about the things he wants to do before his death. (Field notes, telephone call 2 with John)

We were surprised that this was not raised again by John at his second face to face interview. Perhaps he wanted to forget about it; intent on thinking positive, rather than how much time he had left. Or, perhaps the use of the telephone offered him a degree of anonymity when compared with a face to face interview.

The telephone calls often enabled us to identify significant events or changes, but not always. We spoke with Fred every month and usually his wife Jane was at work. Unfortunately, we were unable to meet with Fred for a second interview because he died suddenly after suffering a fall. His wife Jane informed us that Fred had brain metastases, in addition to lung metastases; something which Fred had never mentioned.

### Relationship and reciprocity

The relationship between the researcher and participants in the flexible group evolved in parallel with the telephone calls. Participants in the flexible group often divulged a private account of their experience more readily than those in the routine group – as compared to the public and well-rehearsed cancer story that defined the beginning of the interview. For example, we spoke with Faye every month between interviews 1 and 2. We shared lengthy chats where she told the researcher about her family and friends, in addition to her worries about cancer. At the second face to face interview with Faye she immediately told the researcher that she felt she now had a death sentence.

“I actually feel now, and I never felt it before, I’ve got a death-sentence now.” (Faye, interview 2)

This change could be because she felt more comfortable in the presence of the researcher. This is not to say that those in the 6 monthly interview group did not produce private accounts, rather they told the cancer story before sharing their private accounts, as if first setting the scene.

Although we gained contextual information and sometimes captured real time transitions in the lives of participants in the flexible group, this could have been the result of reasons other than the increased contact and more evolved relationship. For example, some participants were more talkative, particularly the female participants.

### Challenges of the flexible approach to interviewing

#### Balancing ethical integrity and research integrity

This study also explored the feasibility and added value of conducting interviews as close as possible to when change was occurring, but this only happened on two occasions. Eve was told just 2 months after the first interview that she would require a course of the more aggressive chemotherapy and that she had been referred to the hospice for pain control. We were keen to capture this transition to hospice care so we met for an interview. Eve deteriorated quickly after the initial interview, and died quickly after the second interview. Similarly, Faye told the researcher that she had been given a few weeks to live at month 9 of the study and so we invited her to an interview then. Capturing change in real time was challenging because change in the social, psychological and spiritual domains was subtle, and often obvious in retrospect, when we conducted the longitudinal analysis of the individual cases. In contrast, Eve and Faye experienced physical changes and their deterioration was evident.

However, during the study there were times when we felt that conducting a face to face interview was not appropriate, even if there had been a significant change in circumstances. For example, during the penultimate telephone call with Deirdre, she described that the health care team had decided to discontinue treatment. Deirdre was aware that her life was approaching the end and accordingly we respected Deirdre’s priority to be with her family. Needless to say, the same ethical considerations were given to those in the 6 monthly interval group. For example, the third interview with Cath was an informal chat rather than a recorded interview because she was in the hospice and was frail, weak and close to death.

We propose that all the participants would have willingly participated in an interview had they been asked, and it could be argued that deciding not to interview was disempowering them. Given the good relationship that had evolved over the course of the study, perhaps we should have been able to ask them about participating in an interview in such a way that would have allowed them to say no. However, we were concerned that doing so would have threatened the research relationship and ethical integrity of the study. In the cases of Eve and Faye, it could be argued that hastening the interviews in light of their deteriorating condition was unethical, but denying them a voice would have been also. We wanted all the participants to remain autonomous for as long as they could and verbal consent was taken prior to every subsequent interview.

#### The unpredictability of research

South East Scotland Research Ethics Committee had asked us to check the status (alive or deceased) of the participants before making any telephone calls, which we adhered to. However, we were faced with 2 challenging incidents involving the family members of participants from the flexible interval group. First, Fred usually answered the telephone because it was beside his chair in the living room so we were alerted that something was wrong when Jane, his wife answered the telephone. She tearfully explained that Fred had died, peacefully, just days earlier and described the circumstances of his death. Indeed, she had been looking for the researcher’s details so she could call and inform us about what had happened. A similar situation occurred a short while later with Eve’s daughter. These incidents were unique to the flexible group and were a direct result of the increased contact. They highlight the complexity and unpredictability of researching the experiences of people with serious illness, where circumstances change quickly. The outcome of these telephone calls was because of unfortunate timing, and they happened because of limitations in information sharing between health systems.

#### Blurred boundaries

The telephone conversations with participants were often long and profound. Over time, the boundaries of the researcher’s role in maintaining relationships became blurred. This issue was exaggerated in the flexible interval group, which could reflect the more evolved, and involved relationship with the participants. Although the researcher anticipated her role in the project, the same due consideration had not been given to how the participants would perceive the researcher - nurse, confidante, shoulder to cry on - and how this would change as the relationship strengthened and their condition deteriorated. For example, the researcher was asked to comment on the participants’ clinical care. This became more prevalent as the study progressed and the relationship evolved. Although this could be viewed as a positive outcome of the flexible approach and telephone calls, for example a sign of trust; it presented us with a challenge. These were questions that research professionals are not permitted to answer. This was complicated further as the researcher in this study was a nurse and therefore had a basic understanding of participants’ concerns. When such situations occurred we suggested that the participants discuss their concerns with the specialist nurses at the recruiting centre.

## Discussion

In this paper we have presented a practical account of conducting monthly telephone calls in addition to face to face interviews in a qualitative longitudinal study with people towards the end of life. There is currently a dearth of methodological literature relating to qualitative longitudinal methods for health care research and so this article adds to the small, but expanding debate. Qualitative longitudinal research can provide rich insights into the dynamic experience of illness, but as yet it has still to be used to its full potential by health care researchers.

Proponents of qualitative longitudinal research advocate that a responsive approach should be used at all stages in the research process because we do not know what or when change will emerge [1,4]. Understanding change over time is the key tenet of qualitative longitudinal research. The telephone calls allowed us to capture processes, and nuances, that might have otherwise been overlooked, or their significance underestimated, when they were being described retrospectively. This supports Pettigrew’s definition of qualitative longitudinal research, which describes the process of chang*ing* as opposed to change as a one off event [6]. Likewise, Taylor found that the phone calls allowed events to be seen in real time, which could then be discussed at subsequent interviews [29]. We also found that a strength of regular telephone calls was that they informed subsequent interviews, and in this way our findings echo those of Taylor.

The telephone calls enabled greater insight into each of Lewis’s four types of change [5].

1. The first type of change is narrative change and it reflects how the participants’ stories change over time. The contextual information gained through the telephone calls provided a more detailed timeline of events over the course of the study. Moreover, as the relationship with the participants intensified, they shared their private accounts on a more regular basis.
2. The second type of change occurs from the participants’ reinterpretation and retelling of their stories. Those in the flexible group were given more opportunity to retell and refine their stories. We heard about everyday experiences, meaning we captured the small changes, which were often precursors for bigger changes and thus, how the process of change evolved. One of the many advantages of qualitative longitudinal research is that prospective and retrospective accounts of experience can be explored over time [1]. This allowed participants to reflect, recount and recapitulate their experiences as the study progressed. In this way, participants were reminded of events or feelings that they might have otherwise thought insignificant, giving them more detail to reinterpret and retell. This finding is supported by Taylor, who posited that the immediacy of the transitions that the youths in her study experienced had been lost between waves of interviews [29].
3. The third type of change is evoked through the continuous reinterpretation of the researcher. Qualitative longitudinal research encourages reflexivity for the participant and the researcher, and as the study progressed we saw the participants differently as individuals, and in relation to one another. This meant we were continuously reinterpreting their stories. Such reinterpretation was amplified as we developed a reciprocal relationship with the participants, which was particularly the case for participants who were telephoned. As the findings suggest, the data gathered from the telephone calls informed subsequent interviews in a literal way; a participant would tell us something on the phone and we would then probe further about it at the interview. But at another level, the everyday chit chat about their lives influenced the data generation and the analysis in a subtle way.
4. Lewis’s [5] fourth category is no change. In the context of this research, no change was generally positive, because changes usually meant participants were closer to death. Often participants’ chatted about their everyday activities, which supports the idea that is with the normal everyday activities that participants find comfort, control and sense of self [30-36].

### The research relationship

This paper has raised questions about the ethics of the research relationship and highlighted the importance of ensuring the physical and psychological safety of both the researcher and the participant. Qualitative longitudinal research forces the research relationship into starker focus than one-off qualitative studies. Reflexivity is key to rigorous qualitative research and qualitative longitudinal research emphasises the importance of this. The researcher in this study (EC) kept a research diary where she noted her reflections on change – changes in the participant physical and psycho-social wellbeing, changes in her own feelings about the study, participants, interviews or analysis. In this way she could map her own journey with that of the participants. These reflections were included in the longitudinal analyses of the individual cases using diagrammatical timelines to ensure reflexivity.

The researcher’s diary was also used as a way of de-briefing after the interviews and telephone calls. The project did carry an emotional burden and the research team met at regular intervals to discuss the project. The researcher was made aware of counselling services that were available to her and her transcriber. Qualitative longitudinal studies are often thought to emphasis issues concerned with data management, consent, confidentiality, anonymity, ending the participant/researcher relationship [8] and arguably the telephone calls exaggerated this further. However, with careful planning, adequate support and an evidence base of methodological accounts to help qualitative longitudinal researchers, these issues can be overcome.

The participant and the researcher require a trusting relationship which empowers participants to share their stories. There is evidence to suggest that qualitative longitudinal research has therapeutic potential for participants [1,8,29,37-40]. For example, participants talking about diabetes enjoyed the experience and had time to discuss their issues, when compared with rushed hospital appointments [37]. Likewise, parents of children with leukaemia felt it gave them the opportunity to release their thoughts and emotions [39]. The longitudinal aspect allowed participants to reflect on their own stories. Perhaps in doing this they were able make sense of their illness as Frank suggested [18].

### When is increased contact justified?

The findings in this paper suggest that using telephone calls, in addition to face to face interviewing may be indicated to tackle 3 challenges of qualitative longitudinal research.

### Countering attrition

As a result of the telephone calls we conducted 2 interviews that would have otherwise been missed because the participants would have died. Unsurprisingly, attrition is common in qualitative longitudinal studies because of the longitudinal aspect [8]. No participants in this study withdrew, meaning we are unable to say with any certainty that the telephone calls helped to keep participants recruited into the study. Nevertheless, being able to get a sense of what was happening to them in real time meant we could address the small changes that participants experienced. As the study progressed, the telephone calls were useful in keeping up with how people were doing and if they were going to hospital or hospice. The findings in this article largely support Taylor, who found that the frequent contact she had with the vulnerable youths in her study improved retention [29]. Research which involves potentially transient groups of participants might also benefit from regular contact. For example, studies involving participants with mental health issues or addiction, or other groups who are regularly in and out of hospital like those with chronic illness or frail older people.

Strategies such as birthday and Christmas cards, and diaries have been reported in other qualitative longitudinal studies [41-43], but our experience supports Holt, who suggests that telephone calls are a socially inclusive method of generating data [23]. Moreover the telephone calls were informal, and part of what people did in everyday life. This is in contrast to the research interview which none of the participants had undertaken before. Neither were the telephone calls pre-planned in the same way as the face to face interviews which meant we captured what the participants were doing at exactly the time of the phone call. In this way, the telephone calls enabled accounts of a different participant experience to emerge.

### Exploring an unpredictable disease trajectory

Often, the illness trajectory of participants took a sudden unpredictable downward turn. As a result of the regular telephone contact we were able to capture this time. Nissim et al. also incorporated flexibility into their research design to capture how the desire for a hastened death evolved over time for people dying of cancer [44]. Our experience, and that of Nissim et al. supports the recommendation that a flexible approach is necessary in qualitative longitudinal research, because we do not know what change will occur [4].

### Shifting control

Holt found one of the benefits of telephone interviewing to be that participants had control over the privacy of the call, and over their social space [23]. The participants in this study described experiencing a loss of control, and feeling powerless, as they gradually relinquished control to the health professionals who were caring for them. If the telephone calls enabled participants to feel more in control of their social space it might go some way to restoring feelings of empowerment. Sturges and Hanrahan also commented that research participants might be more comfortable discussing sensitive topics on the telephone. In the context of their work ‘sensitive’ meant ‘embarrassing’, but the same could apply to emotionally sensitive topics, such as deciding to stop treatment, choosing where to die or how to involve family in such decisions [45].

### Limitations

This paper reflects the accounts of 16 participants with metastatic colorectal cancer. They were recruited from the same centre within a period of 9 months. It is unlikely that participants’ accounts would have been the same if we had we recruited at a different time, and from a different place. The group were all white Caucasian. Those with moderate to severe memory impairment and those who were considered to be approaching the terminal phase were excluded from the study. No participants withdrew from the study which suggests that the method of recruitment and the qualitative longitudinal approach were acceptable to participants, in an area which is known to be difficult to conduct research.

## Conclusion

Qualitative longitudinal research is increasing in popularity, as researchers appreciate the importance of understanding change in the lives of ill people. As yet, there are inadequate reflections on the qualitative longitudinal methodology itself, particularly within healthcare. This paper has provided a practical account of supplementing face to face longitudinal interviews with telephone calls. There are benefits and challenges to this approach and balancing the research agenda with the ethical agenda, whilst fostering a trusting relationship, was a continuous endeavour. There is agreement that qualitative longitudinal research is about understanding change and adding telephone calls created a more nuanced understanding and greater narrative depth. Moreover, telephone calls between qualitative longitudinal interviews have the potential to overcome some of the complex issues that are inherent to health care research, particularly with those with life-limiting illness. Researchers should not be put off by the challenges, rather embrace the potential for rich, dynamic, contextualised accounts of experience. Finally, we support Calman et al’s plea to encourage qualitative longitudinal researchers to publish their methodological reflections, to move this valuable method forward [10].

## References

[1] Holland J, Thomson R, Henderson S: Qualitative Longitudinal Research: A Discussion Paper. Families and Social Capital ESRC Research Group Working Paper No 21, London South Bank University; 2006, http://www1.lsbu.ac.uk/ahs/downloads/families/familieswp21.pdf. Accessed 10/02/2015

[2] Neale B, Flowerdew J: International Journal of Social Research Methodology 2003, 6: 189-200. doi:10.1080/1364557032000091798

[3] McLeod J, Thomson R (2009). Researching Social Change.

[4] Saldana J (2003). Longitudinal Qualitative Research: Analyzing Change Through Time.

[5] Lewis J (2007). Analysing qualitative longitudinal research in evaluations. Social Policy Society.

[6] Pettigrew A (1990). Longitudinal field research on change: theory and practice. Organization Sci.

[7] Murray SA, Sheikh A (2006). Serial interviews for patients with progressive diseases. Lancet.

[8] Murray SA, Kendall M, Carduff E, Worth A, Harris FM, Lloyd A (2009). Use of serial qualitative interviews to understand patients' evolving experiences and needs. BMJ.

[9] Cavers DMP, Hacking BMD, Erridge SEM, Kendall MMP, Morris PGM, Murray SAM (2012). Social, psychological and existential well-being in patients with glioma and their caregivers: a qualitative study. Can Med Assoc J.

[10] Calman L, Brunton L, Molassiotis A (2013). Developing longitudinal qualitative designs: lessons learned and recommendations for health services research. BMC Med Res Methodol.

[11] Lawton J, Rankin D, Peel E, Douglas M (2009). Patients' perceptions and experiences of transitions in diabetes care: a longitudinal qualitative study. Health Expect.

[12] McGrath P, Paton MA, Huff N (2004). Beginning treatment for paediatric acute myeloid leukaemia: diagnosis and the early hospital experience. Scand J Caring Sci.

[13] Woodgate RL (2006). Life is never the same: childhood cancer narratives. Eur J Cancer Care.

[14] Lowe M, Molassiotis A (2011). A longitudinal qualitative analysis of the factors that influence patient distress within the lung cancer population. Lung Cancer.

[15] Wood JP, Connelly DM, Maly MR (2010). 'Getting back to real living': A qualitative study of the process of community reintegration after stroke. Clin Rehabil.

[16] Crotty M (1998). The Foundations of Social Research: Meaning, and perspective in the research process.

[17] National Cancer Intelligence Network: Colorectal Cancer Survival by Stage - NCIN Data Briefing. http://www.ncin.org.uk/cancer_type_and_topic_specific_work/cancer_type_specific_work/colorectal_cancer/, accessed 05/02/2015 2015.

[18] Frank A (1995). The Wounded Storyteller: Body, Illness and Ethics.

[19] Kleinman A (1988). The Illness Narratives: Suffering, Healing, and the Human Condition.

[20] Mishler EG (1986). Research Interviewing: Context and Narrative.

[21] Mathieson C, Stam H (1995). Renegotiating identity: cancer narratives. Sociol Health Illn.

[22] Hyden LC (1997). Illness and narrative. Sociol Health Illn.

[23] Holt A (2010). Using the telephone for narrative interviewing: a research note. Qual Res.

[24] Richardson L, Denzin N, Lincoln Y (2000). Writing: A method of inquiry. Handbook of Qualitative Research.

[25] Thomson R, Bell R, Holland J, Henderson S, McGrellis S, Sharpe S (2002). Critical moments: choice, chance and opportunity in young People's narratives of transition. Sociol.

[26] Brown L, Gilligan C (1992). Meeting at the Crossroads: Women's psychology and girls development.

[27] Reissman C (1993). Narrative Analysis.

[28] Labov W, Waletzky J. Narrative Analysis: Oral versions of personal experience. In Essays on Verbal and Visual Arts. Edited by Helm J. Seattle: 1967:12-44.

[29] Taylor SA (2009). Engaging and retaining vulnerable youth in a short-term longitudinal qualitative study. Qual Soc Work.

[30] Hubbard G, Kidd L, Kearney N (2010). Disrupted lives and threats to identity: the experiences of people with colorectal cancer within the first year following diagnosis. Health (London).

[31] Shaha M, Cox CL (2003). The omnipresence of cancer. Eur J Oncol Nurs.

[32] Sjovall K, Gunnars B, Olsson H, Thome B (2011). Experiences of living with advanced colorectal cancer from two perspectives - inside and outside. Eur J Oncol Nurs.

[33] Bury M (1982). Chronic illness as biographical disruption. Sociol Health Illn.

[34] Charmaz K (1983). Loss of self: a fundamental form of suffering in the chronically ill. Sociol Health Illn.

[35] Kidd L, Kearney N, O'Carroll R, Hubbard G (2008). Experiences of self-care in patients with colorectal cancer: a longitudinal study. J Adv Nurs.

[36] Rozmovits L, Ziebland S (2004). Expressions of loss of adulthood in the narratives of people with colorectal cancer. Qual Health Res.

[37] Peel E, Parry O, Douglas M, Lawton J (2006). "It's no skin off my nose": why people take part in qualitative research. Qual Health Res.

[38] Lowes L, Paul G (2006). Participants' experiences of being interviewed about an emotive topic. J Adv Nurs.

[39] McGrath P (2003). Benefits of participation in longitudinal qualitative research study. Monash Bioeth Rev.

[40] Dickson-Swift V, James EL, Kippen S, Liamputtong P (2006). Blurring boundaries in qualitative health research on sensitive topics. Qual Health Res.

[41] Inventing Adulthoods: www.lsbu.ac.uk/inventingadulthoods/. 2012.

[42] Thomson R, Holland J, McGrellis S, Bell R, Henderson S, Sharpe S (2004). Inventing adulthoods: a biographical approach to understanding youth citizenship. Sociol Rev.

[43] Goodacre L (2006). Women's perceptions on managing chronic arthritis. Br J Occup Therapy.

[44] Nissim R, Gagliese L, Rodin G (2009). The desire for hastened death in individuals with advanced cancer: A longitudinal qualitative study. Soc Sci Med.

[45] Sturges JE, Hanrahan KJ (2004). Comparing telephone and face-to-face qualitative interviewing: a research note. Qual Res.

